# Abandonment of Prescribed Buprenorphine for Opioid Use Disorder, 2020-2024

**DOI:** 10.1001/jamanetworkopen.2026.27142

**Published:** 2026-08-04

**Authors:** Xinyi Jiang, Kun Zhang, Yijie Chen, Yi-Wen Shih, Nisha Nataraj, Gery P. Guy

**Affiliations:** 1 Division of Overdose Prevention, Centers for Disease Control and Prevention, Atlanta, Georgia

## Abstract

This cohort study analyzes factors associated with abandonment of newly prescribed buprenorphine among US adults from 2020 to 2024.

## Introduction

Buprenorphine is an effective treatment for opioid use disorder (OUD) that reduces mortality risk but remains underutilized.^1^ In 2022, only 1 in 4 US adults who needed medications for OUD (MOUD) received them.^2^ Pharmacy- and patient-level barriers can undermine efforts to expand access to buprenorphine through clinician prescribing.^2,3,4^ Prior research found that 32% of buprenorphine prescriptions written during 2015 to 2019 were not dispensed.^3^ We sought to examine characteristics associated with abandonment of newly prescribed buprenorphine—defined as a prescription that was written but not dispensed or filled—and trends in abandonment among adults during 2020 to 2024 to inform efforts to improve buprenorphine access.

## Methods

This retrospective cohort study used deidentified electronic health records (EHR) data linked with administrative claims from the Optum Labs Data Warehouse. The claims data include commercial and Medicare Advantage (MA) enrollees; the EHR data include individuals receiving care from US participating health care groups and organizations. More details on this data including the linkage method are available elsewhere.^3,5^ We followed the STROBE reporting guideline. Institutional review board review and informed consent were not applicable because deidentified secondary data were used in accordance with 45 CFR §46.

We identified the first prescribed buprenorphine prescription for OUD (prescribing date as index date) of a patient aged 18 years or older between January 1, 2020, and September 25, 2024, in EHR data. With the linked claims data, we included patients who were continuously enrolled with both medical and pharmacy benefits for at least 180 days before the index date and at least 30 days after (with a ≤45-day gap in coverage) (eMethods and eFigures 1-2 in Supplement 1).

The outcome, buprenorphine prescription abandonment, was defined as the lack of dispensing or filling of the first prescribed buprenorphine (ie, no dispensing records in claims data) within 30 days of the index date, including prescriptions not filled by patients or rejected by insurers or pharmacies. We compared the distribution of characteristics among patients with buprenorphine prescriptions dispensed vs abandoned using *t* tests for continuous variables and Pearson χ^2^ tests for categorical variables. Race and ethnicity from EHR data were self-reported by patients or observed by clinicians and was included to account for racial disparities in overdose and OUD treatment. We used weighted least-squares regression to examine linear trends in the yearly proportion of patients with an abandoned first buprenorphine prescription during 2020 to 2024. A 2-sided *P* < .05 was considered statistically significant. All analyses were conducted using SAS version 9.4 (SAS Institute).

## Results

The final cohort included 1428 patients with a first buprenorphine prescription for OUD (mean [SD] age, 56.6 [14.7] years; 723 male [50.6%]; 113 Black [7.9%]; 90 Hispanic [6.3%]; 1199 White [84.0%]) (Table). Among all patients, 369 (25.8%) had buprenorphine prescription abandonment and 1059 (74.2%) did not. Patients with abandoned prescriptions were younger (mean [SD] age, 54.3 [16.2] vs 57.4 [14.1] years), were more frequently Hispanic (39 patients [10.6%] vs 51 patients [4.8%]), were more likely to have commercial insurance (191 patients [51.8%] vs 311 patients [29.4%]), and had higher pharmacy deductibles (mean SD, $483.80 [$1104.50] vs $296.40 [$712.30]) than those dispensed buprenorphine. Compared with patients with abandoned prescriptions, patients dispensed buprenorphine had a higher percentage of OUD or overdoses (576 patients [54.4%] vs 148 patients [40.1%]), other substance use disorder diagnoses (222 patients [21.0%] vs 57 patients [15.5%]), or mental health diagnoses (556 patients [52.5%] vs 142 patients [38.5%]) 180 days before or on the index date. No statistically significant differences in sex, race, or tier-2 retail pharmacy copay were identified between groups. The proportion of first prescriptions being abandoned increased from 19% in 2020 to 37% in 2024 (*P* for trend = .02) (Figure).

**Table.** Characteristics of Adult Patients With the First Buprenorphine Prescription for Treating Opioid Use Disorder by Prescription Abandonment Status, January 1, 2020, to September 25, 2024

Patient characteristics at index date	Patients, No. (%)			*P* value
	Total (N = 1428)^a^	Buprenorphine prescription dispensed within 30 d (n = 1059)^b^	Buprenorphine prescription abandonment within 30 d (n = 369)	
Age, mean (SD), y	56.6 (14.7)	57.4 (14.1)	54.3 (16.2)	.001
Sex				
Female	705 (49.4)	526 (49.7)	179 (48.5)	.70
Male	723 (50.6)	533 (50.3)	190 (51.5)	
Race^c^				
Black	113 (7.9)	89 (8.4)	24 (6.5)	.52
White	1199 (84.0)	912 (86.1)	287 (77.8)	
Other or unknown	116 (8.1)	58 (5.5)	58 (15.7)	NA
Ethnicity^c^				
Hispanic	90 (6.3)	51 (4.8)	39 (10.6)	.003
Not Hispanic	1239 (86.8)	956 (90.3)	283 (76.7)	
Other or unknown	99 (6.9)	52 (4.9)	47 (12.7)	NA
Census region				
Midwest	258 (18.1)	190 (17.9)	68 (18.4)	.002
Northeast	133 (9.3)	81 (7.7)	52 (14.1)	
South	713 (49.9)	544 (51.4)	169 (45.8)	
West	252 (17.7)	184 (17.4)	68 (18.4)	
Other or unknown	72 (5.0)	60 (5.7)	12 (3.3)	NA
Insurance type				
Commercial	502 (35.1)	311 (29.4)	191 (51.8)	<.001
Medicare Advantage	926 (64.9)	748 (70.6)	178 (48.2)	
Cost-sharing in pharmacy benefit design				
Tier 2 retail pharmacy copay, mean (SD), $^d^	19.90 (15.00)	19.30 (14.20)	21.60 (17.00)	.05
Pharmacy deductibles, mean (SD), $^e^	342.50 (829.90)	296.40 (712.30)	483.80 (1104.50)	.006
Calendar quarter of the index date				
1	446 (31.2)	349 (33.0)	97 (26.3)	.008
2	321 (22.5)	246 (23.2)	75 (20.3)	
3	361 (25.3)	260 (24.6)	101 (27.4)	
4	300 (21.0)	204 (19.3)	96 (26.0)	
Clinical characteristics 180 d before or on the index date				
Opioid use disorder or overdoses	724 (50.7)	576 (54.4)	148 (40.1)	<.001
Any other substance use disorder diagnosis^f^	279 (19.5)	222 (21.0)	57 (15.5)	.02
Any mental health diagnosis^g^	698 (48.9)	556 (52.5)	142 (38.5)	<.001

Abbreviation: NA, not applicable.

^a^ Among 1428 patients with the first buprenorphine prescription, 1415 (99.1%) had prescriptions for oral buprenorphine (including sublingual or buccal).

^b^ The mean (SD) time between the index date and the date of buprenorphine prescription dispensed within 30 days of the index date was 1.1 (3.9) days.

^c^ Race and ethnicity were self-reported by patients or as observed by the clinician. Other or unknown race included Asian and any racial group not otherwise specified. The other or unknown ethnicity category was created by the Optum Labs Data Warehouse (OLDW), and there is no way to differentiate other or unknown in the OLDW electronic health record database.

^d^ Overall, 979 of 1428 patients (68.6%) had tier 2 pharmacy copay information, 717 of 1059 (67.7%) had tier 2 pharmacy copay information in the dispending group, and 262 of 369 (71.0%) had tier 2 pharmacy copay information in the abandonment group. Pharmacy copay for tier 2 retail drug was included for the period from 180 days before the index date to 30 days after the index date. We chose the tier 2 pharmacy copay because most generic buprenorphine products for treating opioid use disorder were categorized as tier 2.

^e^ Overall, 1250 of 1428 patients (87.5%) patients had deductibles information, 942 of 1059 (90.0%) had deductibles information in the dispending group, and 308 of 369 (83.5%) had deductibles information in the abandonment group. Maximum deductible in pharmacy benefit design for both dispensing and abandonment groups was $7000. Deductibles were included for the period from 180 days before the index date to 30 days after the index date.

^f^ Included alcohol use disorder, cannabis use disorder, cocaine use disorder, sedative and hypnotic related disorder, stimulant use disorder, and other psychoactive substance use disorder.

^g^ Included anxiety, major depression, attention-deficit/hyperactivity disorder, posttraumatic stress disorder, schizophrenia, and other mood disorders including bipolar.

**Figure.**
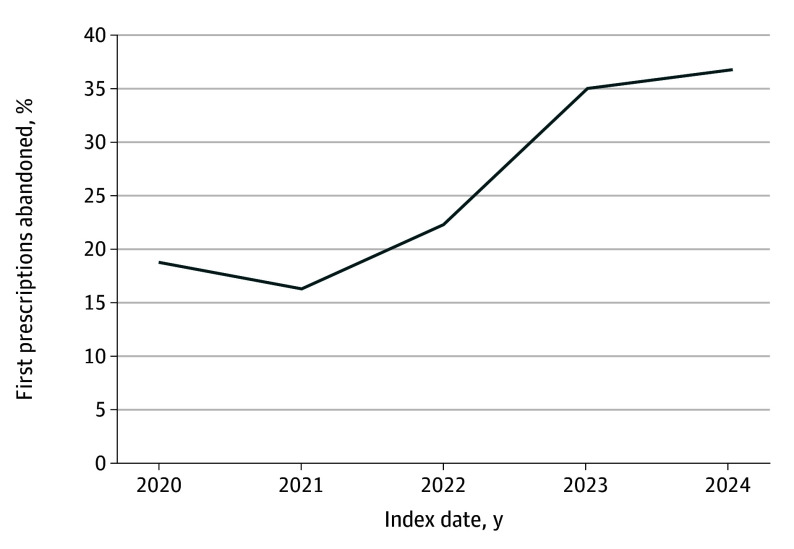
Trends in First Buprenorphine Prescription Abandonment, January 1, 2020, to September 25, 2024 Authors’ analysis of electronic health record–claims linked datasets from Optum Labs Data Warehouse between January 1, 2020, and September 25, 2024. A weighted least-squares regression was used to examine linear trends in the yearly proportion of patients with an abandoned first buprenorphine prescription for opioid use disorder from 2020 to 2024. The proportion of the first prescriptions being abandoned increased from 19% in 2020 to 37% in 2024 (*P *for trend = .02).

## Discussion

This cohort study found that the proportion of abandoned newly prescribed buprenorphine prescriptions for OUD increased from 2020 to 2024. Over 1 in 3 patients’ first buprenorphine prescriptions were abandoned in 2024, representing a missed opportunity for treatment initiation and engagement.

Hispanic patients had a higher proportion of prescription abandonment, possibly due to language^6^ and pharmacy-level barriers, such as limited retail pharmacies in Hispanic communities and neighborhoods.^7^ Increasing opioid overdose death rates among Hispanics^8^ highlights the importance of expanding access among this population.

Prior research found limited associations of overall cost-sharing with buprenorphine prescription abandonment, without distinguishing among types of cost-sharing.^9^ Our findings suggest that pharmacy plan deductibles may be an important factor associated with abandonment. Additionally, we found a higher proportion of buprenorphine prescription abandonment among commercial vs MA enrollees. Prior research showed various progress with reducing buprenorphine coverage restrictions among different insurers and called for continuously monitoring buprenorphine coverage barriers across all insurer types.^10^ Our finding warrants further analysis to help understand the role of insurance benefits design (ie, prior authorization, quantity limits, and cost-sharing).

Study limitations include limited generalizability beyond the commercial and MA population, inability to distinguish prescriptions not filled by patients from those rejected by insurers or pharmacies, and potential misclassification of abandonment among prescriptions paid through cash, assistance programs, or other insurance plans not captured in linked claims data.

Policy changes, including removal of waiver-related training requirements^4^ and pharmacy benefit design, may not sufficiently address access barriers. Public health efforts, such as directly dispensing buprenorphine during clinical encounters,^3^ strengthening linkage to care, and improving buprenorphine availability in pharmacies, could help reduce barriers to treatment access and reduce overdoses.

## Notes

Supplement 1. eMethods. Buprenorphine Identification eFigure 1. Study Design eFigure 2. Flow Chart eReferences

Supplement 2. Data Sharing Statement
