# Supplementary material for: Abandonment of Prescribed Buprenorphine for Opioid Use Disorder, 2020-2024
Source: JAMA Netw Open. 2026 Aug 4;9(8):e2627142. doi: 10.1001/jamanetworkopen.2026.27142 (PMC13439425; doi:10.1001/jamanetworkopen.2026.27142)
Supplement: Supplementary file 1 [file jamanetwopen-e2627142-s001.pdf]

Supplemental Online Content

Jiang X, Zhang K, Chen Y, Shih YW, Nataraj N, Guy GP. Abandonment of prescribed buprenorphine for opioid use disorder. *JAMA Netw. Open*. 2026;9(8):e2627142. doi:10.1001/jamanetworkopen.2026.27142

eMethods. Buprenorphine Identification

eFigure 1. Study Design

eFigure 2. Flow Chart

This supplemental material has been provided by the authors to give readers additional information about their work.

© 2026 Jiang X et al. *JAMA Network Open*.

eMethods. Buprenorphine Identification

Prescriptions: Prescribed buprenorphine for opioid use disorder (OUD) was identified from the prescriptions table in the Optum Labs Data Warehouse (OLDW) electronic health records (EHR) data by generic names containing buprenorphine.

Dispensing: Buprenorphine prescriptions dispensed for OUD were identified from OLDW pharmaceutical claims (including Part D for Medicare Advantage) using National Drug Codes and from medical claims using procedure codes[1] for buprenorphine injection.

Buprenorphine products indicated for pain management were excluded.

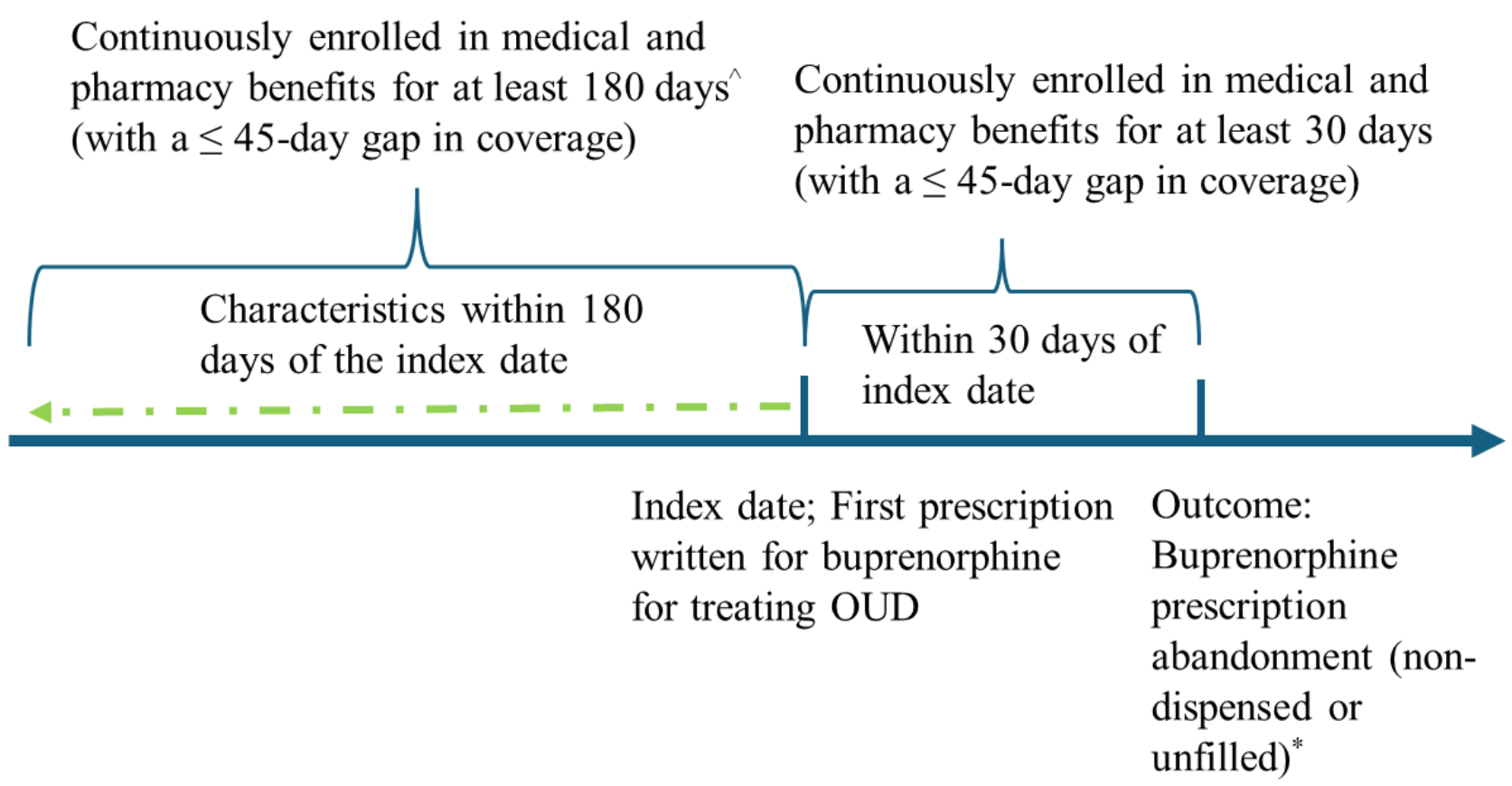

eFigure 1. Study Design

Abbreviation: OUD, opioid use disorder.

^ Authors' analysis of EHR-claims linked datasets from Optum Labs Data Warehouse between January 1, 2020, and September 25, 2024. Data from 2019 were used as a run-in period to identify patients with at least 180-day continuous enrollment before their index date in early 2020.

* National Drug Codes for buprenorphine prescriptions dispensed for OUD are available upon email request from the corresponding author.

© 2026 Jiang X et al. *JAMA Network Open*.

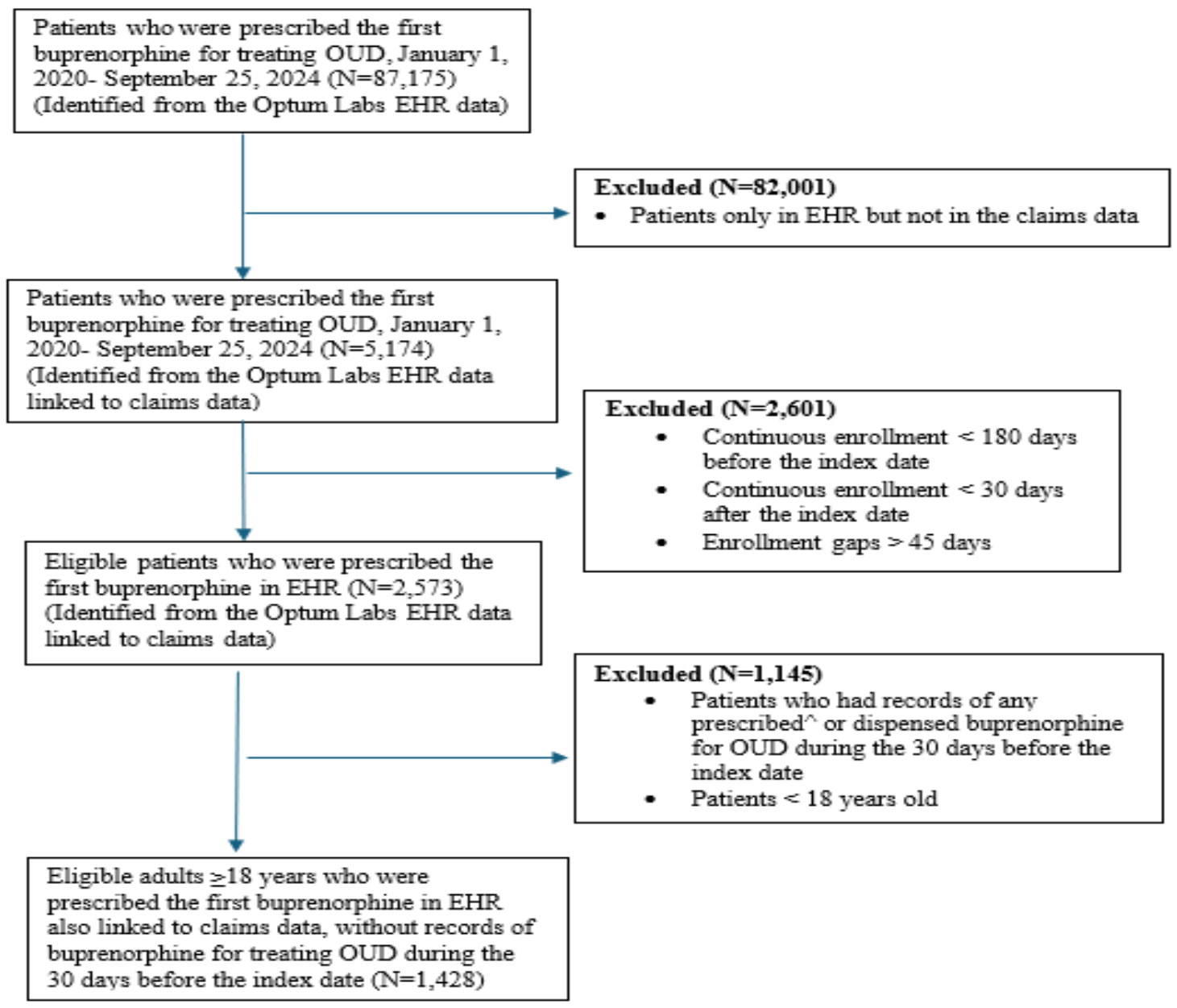

eFigure 2. Flow Chart

Abbreviations: EHR, electronic health record; OUD, opioid use disorder.

^ Patients who received the first buprenorphine prescription in early 2020 may have prescribed buprenorphine during the 30 days before the index date in 2019. We thus excluded these patients. We used 30 days because the mean days' supply of buprenorphine prescription was less than 30 days.[2]

eReferences

1. Reimbursement for Medications for Addiction Treatment Toolkit. https://sitefinitystorage.blob.core.windows.net/sitefinity-production-blobs/docs/default-source/advocacy/reimbursement-for-mat-toolkit-060122.pdf?sfvrsn=80246fce_3

2. Strahan AE, Desai S, Zhang K, Guy GP. Trends in Out-of-Pocket Costs for and Characteristics of Pharmacy-Dispensed buprenorphine Medications for Opioid Use Disorder Treatment by Type of Payer, 2015 to 2020. JAMA Network Open. 2023;6(2):e2254590. doi:10.1001/jamanetworkopen.2022.5459

© 2026 Jiang X et al. *JAMA Network Open*.
