# Supplementary material for: Abandonment of Prescribed Buprenorphine for Opioid Use Disorder, 2020-2024
Source: JAMA Netw Open. 2026 Aug 4;9(8):e2627142. doi: 10.1001/jamanetworkopen.2026.27142 (PMC13439425; doi:10.1001/jamanetworkopen.2026.27142)
Supplement: Supplementary file 2 [file jamanetwopen-e2627142-s002.pdf]

Data Sharing Statement

Jiang. Abandonment of Prescribed Buprenorphine for Opioid Use Disorder, 2020-2024. *JAMA Netw Open*. Published August 04, 2026. doi:10.1001/jamanetworkopen.2026.27142

Data
Data available: No. Optum Labs data are proprietary and only available for purchase by contacting Optum Labs.
